# Engineered bacteriophages for therapeutic and diagnostic applications

**DOI:** 10.1242/dmm.052393

**Published:** 2025-09-30

**Authors:** Kandas Traore, Damien Seyer, Agnes Mihajlovski, Antonia P. Sagona

**Affiliations:** ^1^ CY Cergy Paris Université, ERRMECe Laboratory, Biomaterials for Health Group, Neuville Sur-Oise 95031, France; ^2^ School of Life Sciences, Warwick University, Gibbet Hill Campus, Coventry CV4 7AL, UK

**Keywords:** Bacteriophages, Genomic engineering, Antibiotic resistance, Nanomedicine

## Abstract

Antimicrobial resistance represents one of the most serious threats to both public health and economic sustainability. One of the promising approaches to address this problem is phage therapy – treatment of pathogenic bacterial infections using bacteriophages. Bacteriophages have a narrow host spectrum of activity, minimal side effects and self-replication at the infection site, which positions them as promising candidates to complement or replace conventional antibiotics. Moreover, they can be easily genetically modified to enhance their effectiveness and safety. In this At a Glance article, we highlight the timely relevance of engineered phages as an innovative solution in a rapidly evolving healthcare landscape. First, we introduce bacteriophages' life cycle, ecology and therapeutic history, emphasizing their role in One Health strategies. Then, we describe advanced engineering techniques that can be used to expand bacteriophages' functionalities. Finally, we discuss innovative applications of engineered bacteriophages in biotechnological applications and as a potential countermeasure for antimicrobial resistance, including serving as a shuttle for delivering genes and drugs to the targeted bacterial and eukaryotic cells, targeting intracellular bacteria, contributing to vaccine development, facilitating advancements in tissue engineering and improving bacteriophages' antibacterial properties.

## Teaser

**Summary:** This At a Glance article highlights how engineered bacteriophages can be tailored to enhance antibacterial therapies and diagnostics and to support innovative biomedical applications beyond antimicrobial resistance challenges.

**Figure:**
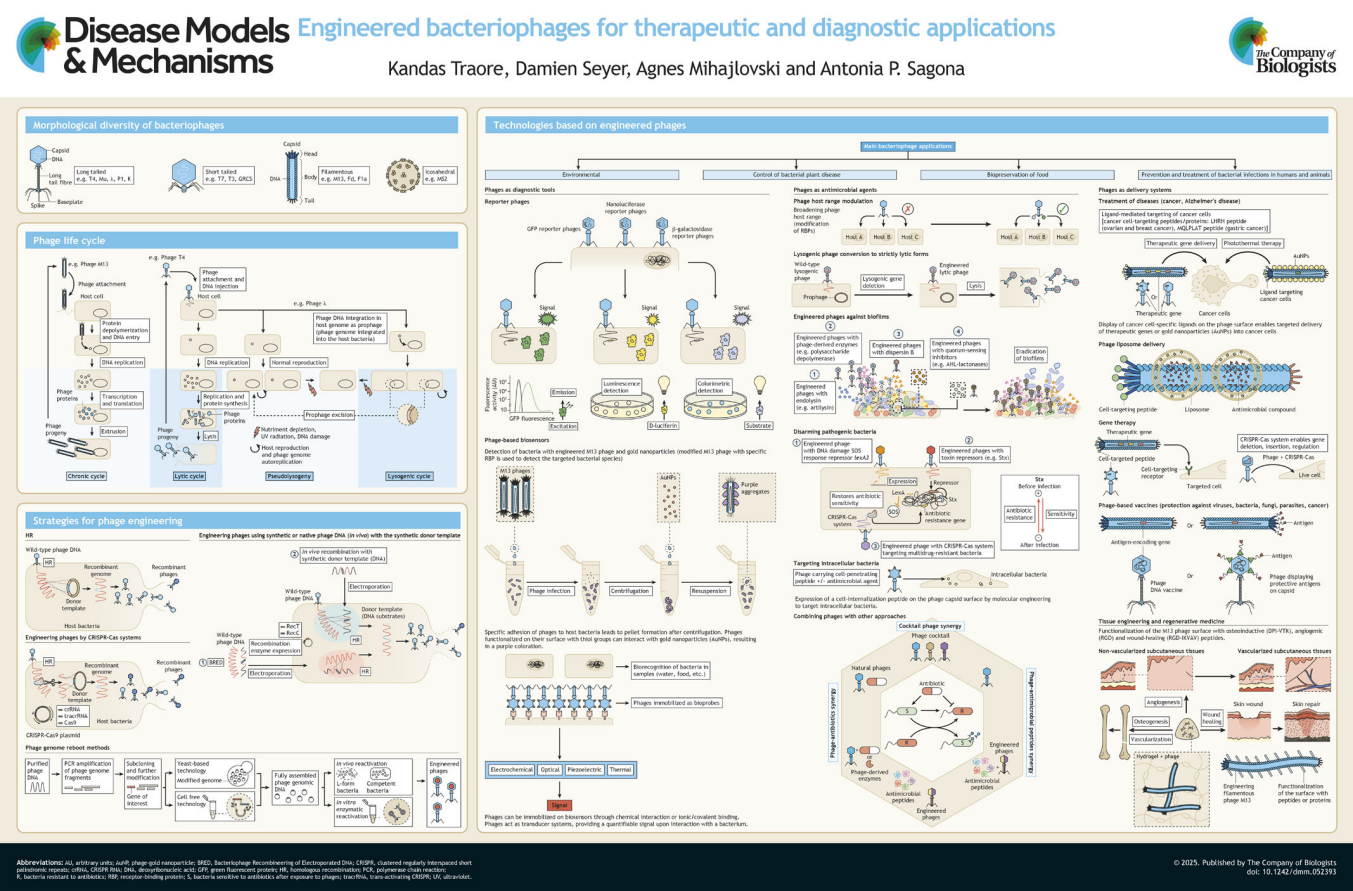
See supplementary information for a high-resolution version of the poster.

## Introduction

The discovery and use of antimicrobial agents to treat infections dates to ancient civilizations, when natural extracts were utilized for their therapeutic properties. Although the concept of employing microorganisms against other microbes has deep historical roots, the accidental discovery of penicillin by Alexander Fleming in 1928 marked the beginning of modern antibiotic therapy (Fleming, 1929). However, the widespread and often inappropriate use of antibiotics, such as for viral infections or in livestock for growth promotion, has led to a major global health threat: antibiotic resistance, the ability of bacteria to survive and grow despite exposure to antibiotics (Gould, 2016; Muteeb et al., 2023). As a result, infections that were once easily treatable have become increasingly hard to manage, resulting in extended hospital stays, increased healthcare costs and elevated mortality rates (Christensen, 2021). Before antibiotics became widespread, bacteriophages (or phages), viruses that infect bacteria, had already been investigated as potential antibacterial agents. The therapeutic use of phages began in the early 20th century, with reports of antibacterial activity in river waters by Hankin (1896), and later observations by Twort (1915) and d'Herelle (1917), who applied phages to treat children suffering from dysentery (d'Herelle, 1931). While research on phage therapy continued in Eastern Europe and the former Soviet Union, Western countries shifted focus to antibiotics following their development in 1940 (Abedon et al., 2011). Nevertheless, the mid-20th century marked the ‘golden age’ of phage research, as phages became essential tools for elucidating key processes such as DNA replication and genetic regulation, shaping the progress of molecular biology (Marinelli et al., 2008; Keen, 2015).

To overcome limitations of natural phages, which include a narrow host range, instability (e.g. sensitivity to immune clearance) and natural development of antimicrobial resistance, various molecular engineering approaches have been developed. One of the earliest molecular tools applied to phages was phage display (Smith, 1985), which enabled the presentation of peptides or proteins on phage capsids and enabled the study of receptor binding and protein–protein interactions. Later, the development of homologous recombination (HR) techniques (Murphy, 2012) provided more precise genetic tools for engineering phages, enabling functional studies of phage proteins and host specificity (Citorik et al., 2014). More recent technologies – including clustered regularly interspaced short palindromic repeats (CRISPR)-Cas-based editing, which allows efficient selection of recombinant phages; *de novo* genome synthesis, which allows the assembly of entirely synthetic or modified genomes; and rebooting of infectious phages in L-form cells, which enables engineering of phages infecting Gram-positive hosts by bypassing transformation barriers – significantly expanded the scope of phage engineering. These approaches have allowed the modification of phages to improve their antimicrobial efficacy, broaden their host range, deliver specific genes or drugs, or serve as diagnostic platforms (Kilcher and Loessner, 2019; Jiang et al., 2022). The recent rise in antimicrobial resistance poses an increasing health threat. In 2019, it was directly responsible for an estimated 1.27 million deaths worldwide (Murray and Ikuta, 2022). Without effective global action, this number could rise to 10 million deaths annually by 2050 (O'Neill, 2016). This growing burden, combined with advances in genome editing, synthetic biology and DNA synthesis technologies, has led to a renewed interest in bacteriophages, not only as natural antibacterial agents, but also as engineered tools for therapeutic and biotechnological applications.

In this At a Glance article, we focus specifically on recent progress in the molecular engineering of bacteriophages and their emerging applications. The goal of this article is to provide an overview of the tools and methods currently used to modify phages and to illustrate how engineered phages can contribute to therapeutic strategies, diagnostics and nanotechnology. We also discuss current challenges in the field and potential ways to address them.

## Bacteriophages: infection cycles, ecological roles and use in therapeutics

Bacteriophages are the most abundant biological entities on Earth, exhibiting extraordinary genetic diversity and structural variability (Campbell, 2003). Their morphology ranges from tailed to filamentous and icosahedral forms. Tailed phages, recently classified under *Caudoviricetes*, make up over 96% of known bacteriophages (Turner et al., 2023). Based on morphology, tailed phages are commonly divided into three main groups: those with long contractile tails (*Myoviridae*), those with long non-contractile tails (formerly *Siphoviridae*) and those with short non-contractile tails (*Podoviridae*) (see poster, ‘Morphological diversity of bacteriophages’). Although these traditional families are no longer recognized in official taxonomy, they remain useful for describing virion structure. Other phage morphologies include filamentous forms, such as those in the *Inoviridae*, which typically cause chronic infections, and icosahedral tailless phages such as *Microviridae* and *Tectiviridae* (Turner et al., 2023).

Bacteriophages infect bacteria by recognizing specific bacterial surface receptors (e.g. outer membrane protein A, lipopolysaccharide, teichoic acid, pili) through receptor-binding proteins (RBPs). To reach and expose these surface receptors, depolymerases produced by phages degrade protective capsular polysaccharides and biofilm exopolysaccharides, facilitating phage attachment to bacteria (Leprince and Mahillon, 2023). Once bound, phages inject their DNA into the bacterial cytoplasm and redirect bacterial replication and expression machinery to replicate the phage genome and synthesize phage proteins, leading to the production of new phage virions (see poster, ‘Phage life cycle’).

Depending on their preferred life cycle, bacteriophages are divided into virulent, temperate and chronic phages (see poster, ‘Phage life cycle’). In the lytic cycle, characteristic of virulent phages, mature particles are released through host cell lysis mediated by holins and endolysins that degrade the plasma membrane and peptidoglycan layer, respectively (Stone et al., 2019). Conversely, temperate phages employ lysogeny, integrating their genome into the host genome and remaining as dormant prophages. Environmental stress (e.g. nutriment depletion, UV radiation, DNA damage) can induce prophages to exit the host genome, transitioning from the lysogenic to the lytic cycle (Huber and Waldor, 2002; Stone et al., 2019). Some phages follow chronic infection cycles, characterized by the persistent release of phage progeny from an infected host without inducing host cell lysis. Instead, phages exit the cell either by pinching off portions of the host membrane (budding) or by being secreted through specialized secretion systems (extrusion) (Stone et al., 2019). Lastly, persistent infections, such as pseudolysogeny and the phage-carrier state, involve phage replication within a subpopulation of host cells. In the phage-carrier state, infected cells continuously produce and release phage particles, while remaining metabolically active and dividing, maintaining a stable coexistence with the phage without undergoing lysis. These persistent infection states are often associated with reduced phage infection efficiency, owing to factors such as receptor saturation (when binding sites on the bacterial surface are occupied or unavailable), enzymatic modification or degradation of receptors, or superinfection immunity (a mechanism by which lysogenized bacteria resist subsequent infection by the same or related phages). Under certain environmental conditions, including nutrient limitation or host stress, these phages may transition into a lytic cycle, periodically disrupting host populations and maintaining dynamic co-existence within microbial communities (Weinbauer, 2004).

Beyond individual infections, phages play a crucial ecological role, regulating microbial populations and promoting genetic exchange. The co-evolution of phages and bacteria drives a continuous arms race between them. Bacteria develop antiphage defences such as receptor modification, whereby surface binding sites are altered or masked to block phage attachment; CRISPR-Cas systems, which provide adaptive immunity by targeting and degrading phage DNA; and abortive infection, a mechanism by which the infected cell self-destructs to prevent phage replication and protect the population (Hampton et al, 2020). In turn, phages evolved counterstrategies, which include altered receptor binding, whereby mutations allow phages to recognize modified or alternative bacterial surface receptors; the production of depolymerases, enzymes that degrade protective bacterial structures such as capsules or biofilms; anti-CRISPR proteins, which directly inhibit the bacterial CRISPR-Cas immune system; and restriction site modification, which enable phages to evade cleavage by host restriction enzymes by altering or masking recognition sites in their DNA (Meyer et al., 2012; Bondy-Denomy et al., 2016). The dynamic co-evolutionary arms race between phages and bacteria is crucial for understanding phage ecology and for developing biotechnological applications, including biocontrol in agriculture and food production, as well as phage therapy. Phage specificity enables precise targeting only of pathogenic bacteria while preserving the beneficial microbiota of the host, such as the gut and skin microbiome, and limiting the development of bacterial resistance. These properties have prompted the renewed interest in phage therapy as a strategy against multidrug-resistant pathogens, especially those in the ESKAPE group (Box 1) (MacNair et al., 2024). Phages also represent a promising tool within the One Health frameworks and are used in food safety, agriculture and environmental applications (Gamachu et al., 2022).

Box 1. One Health framework and ESKAPE group of pathogens The need to discover new antibiotics for bacterial infections has never been more urgent. Paradoxically, few pharmaceutical companies are developing novel antibiotics owing to difficulties in clinical development and associated scientific, regulatory and economic issues. According to the World Health Organization, the ‘ESKAPE’ group of pathogens – *Enterococcus faecium*, *Staphylococcus aureus*, *Klebsiella pneumoniae*, *Acinetobacter baumannii*, *Pseudomonas aeruginosa* and *Enterobacter* species – are at the centre of the fight against antimicrobial resistance (Daruka et al., 2025) owing to their rapid resistance mechanisms against conventional antibiotics and their major contribution to hospital-acquired infections. In 2019, between 1 and 5 million deaths were associated with bacterial antibiotic resistance, and, if current trends continue, this could lead to 10 million deaths per year by 2050 (Murray and Ikuta, 2022). These pathogens' ability to escape the effects of antimicrobials underscores the urgency for comprehensive control strategies. The ‘One Health’ concept provides an essential framework for addressing this challenge, recognizing the intrinsic interrelationship of human, animal and environmental health. Beyond healthcare settings, ESKAPE pathogens are widely distributed across diverse reservoirs, including livestock, companion animals, wildlife, and various environmental niches such as wastewater, soil and food production systems (Daruka et al., 2025). This ubiquitous presence contributes to the dissemination of antibiotic resistance genes across diverse sectors. Consequently, effective management of ESKAPE pathogens demands a multidisciplinary and collaborative One Health approach. This involves coordinated surveillance, prevention and intervention efforts spanning human medicine, veterinary medicine and environmental science. Ultimately, integrating these perspectives is crucial for understanding the epidemiology of ESKAPE pathogens and for developing sustainable and innovative solutions to mitigate their global health impact.

A major limitation for the broader application of phage therapy in clinics, which relies on bacteriolytic phages, is bacterial resistance arising from phage–bacteria co-evolution (Hasan and Ahn, 2022). One approach to addressing this challenge is directed evolution, which involves iterative phage propagation (repeated rounds of phage infection and selection to enhance their ability to infect resistant bacteria) on bacterial hosts to enhance phage efficacy by promoting desired phenotypes or genotypes (Burrowes et al., 2019). Because phage therapy can increase the susceptibility of bacteria to antibiotics (Tadesse et al., 2025), combined use of phage therapy with antibiotics has resulted in improved survival rates. Indeed, clinical case reports have reported improved outcomes in patients with multidrug-resistant infections treated with combined phage–antibiotic therapy. For example, data cited by Diallo and Dublanchet (2022) describe a 97% eradication rate of dysenteric bacilli colonization with phage–antibiotic combination, compared to 83% with antibiotics alone. Similarly, a study conducted in Nigeria reported an increase in survival from 55% to 80% in patients receiving combined therapy (Uchechukwu and Shonekan, 2024). Furthermore, the rapid bactericidal activity, broader host range and low levels of resistance associated with phage-derived enzymes (Liu et al., 2023) and antimicrobial peptides (Mirski et al., 2019; Gouveia et al., 2022) make them an interesting adjuvant to phage therapy, as previously reported (Wang et al., 2023).

Despite their intrinsic specificity and potent antibacterial activity, the clinical use of bacteriophages remains limited by factors such as narrow host range (Álvarez-Espejo et al., 2024), emergence of bacterial resistance (Hasan and Ahn, 2022), reduced efficacy in biofilms (Wang et al., 2023) and sensitivity to immune clearance (Pirnay, 2020). In addition, challenges related to production, formulation and delivery complicate their therapeutic application (Ahmad et al., 2024; Kapoor et al., 2024; Fowoyo, 2024). Addressing these limitations requires engineering strategies aimed at modifying phage properties, including host range, resistance to bacterial defence mechanisms and delivery efficiency. The following section outlines key approaches in phage engineering developed to enhance their biomedical and biotechnological potential.

## Bacteriophage engineering approaches

Phages are viruses composed of proteins and nucleic acids that specifically infect bacteria and are generally considered safe for humans, although their therapeutic use may require attention to immunogenicity and formulation aspects (Pirnay, 2020). They can be modified or enhanced through genetic engineering. Owing to their unique size and shape, engineered phages have broad applications across material science, medicine and nanotechnology (Jiang et al., 2022). The resurgence of phage research, often termed the ‘phage renaissance’, has advanced molecular biology and innovative antimicrobial strategies, such as the development of engineered lysis-deficient phages, phage–antibiotic combination therapies and CRISPR-based precision phage constructs (Kapoor et al., 2024). The convergence of genomics and synthetic biology has deepened our understanding of phage biology, paving the way for rapid and precise genome engineering approaches (Lewis et al., 2024), crucial for expanding therapeutic and biotechnological applications.

Among these engineering strategies, phage display technology stands out as a versatile platform for both selecting high-affinity binders and engineering functionalized phages. This technique, which earned George P. Smith and Sir Gregory P. Winter the 2018 Nobel Prize in Chemistry, relies on the genetic modification of phages to display peptides or proteins on their coat proteins (the structural proteins forming the phage capsid). These engineered phages are then screened against a target of interest, allowing for the selection of high-affinity binders (Jaroszewicz et al., 2022). Through phage bio-panning (an iterative selection method involving repeated cycles of binding to the target, washing away non-binders, elution of bound phages and amplification in bacteria) and the use of highly diverse phage libraries, phage display enables the identification of target-specific peptides and antibodies (Jiang et al., 2022). Beyond molecule discovery, phage display supports the development of sensitive diagnostic assays and ligand screening for disease biomarkers and has been broadly applied in fields such as therapeutic antibody discovery (Zhang, 2023), targeted drug delivery (André et al., 2022) and biomaterials engineering (Davidson et al., 2020). Several of these applications will be further discussed in this article, particularly in the ‘Engineered bacteriophages as diagnostic tools’ and ‘Engineered bacteriophages as nano-biomaterials for eukaryotic applications’ sections.

One of the earliest tools in phage engineering is HR (see poster, ‘Strategies for phage engineering’), a natural DNA repair mechanisms that uses homologous sequences to repair double-stranded DNA breaks (Sharan et al., 2009). In phage engineering, HR enables the generation of gene insertions, deletions or modifications within their bacterial hosts (Pines et al., 2015). This involves introducing a donor DNA matrix (DM) flanked by sequences homologous to the target region of the phage genome into a plasmid, which is then transferred into the host bacteria. During phage replication, recombination between the phage genome and the DM can result in the release of recombinant phages with desired modifications (Mahler et al., 2023). One limitation of the HR approach is the low frequency of successful recombination (<1%), which requires extensive screening.

This limitation can be mitigated by the heterologous expression of recombination enzymes (e.g. Che9c RecET-like proteins) in the bacterial host. These enzymes, expressed from a plasmid, catalyze HR by facilitating strand invasion and exchange using homologous DNA sequences (see poster, ‘Strategies for phage engineering’) (van Kessel et al., 2008). For instance, the Bacteriophage Recombineering of Electroporated DNA (BRED) method facilitates production of recombinant phage genomes by co-electroporating purified phage DNA and a synthetic DM containing the desired mutation into bacterial cells expressing the recombination enzymes. This increases the native HR rate and releases more modified virions (Wetzel et al., 2021). However, the method also presents limitations. First, BRED is limited by the size of the phage genome; phages with large genomes do not transfect bacterial hosts efficiently, which makes the recovery of recombinants much more difficult. Second, recombination efficiency varies widely depending on the nature of the genetic modification. Whereas small deletions can produce recombinants at moderate frequencies (4-60%), larger deletions, insertions or gene replacements are recovered much less efficiently (<1%) (Wetzel et al., 2021).

These limitations can be overcome by using the CRISPR-Cas system. CRISPR-Cas-mediated genome engineering can be applied either after HR, as a counter-selection system to eliminate phages that carry unwanted (wild-type) sequence, or simultaneously with HR to enhance its efficiency (see poster, ‘Strategies for phage engineering’). Most CRISPR-Cas systems used for phage engineering involve the combination of a guide RNA, which matches a target sequence, with an endonuclease Cas (Driehuis and Clevers, 2017). The guide RNA directs the Cas to the target site, where it induces a double-stranded DNA break in wild-type phage genomes. When applied after HR, CRISPR-Cas selectively cleaves non-recombined (wild-type) genomes (counter-selection), thereby enriching for recombinant phages (Kilcher and Loessner, 2019). Alternatively, when used in conjunction with HR, the double-stranded break introduced by CRISPR-Cas systems can stimulate homology-directed repair, facilitating precise sequence modifications (Duong et al., 2020). In both cases, this system contributes to the efficient selection or generation of recombinant phages. Moreover, CRISPR-Cas systems allow for multiplex editing, enabling simultaneous modifications at multiple genomic loci (Kanafi and Tavallaei, 2022). This approach was recently applied to *Pseudomonas aeruginosa* phages using a Cas12a system, in which dual guide RNAs led to efficient editing of two genomic targets within a single step (Chen et al., 2024). Despite the advantages of using CRISPR-Cas systems for phage engineering, several limitations remain. CRISPR-Cas systems require the presence of a short recognition sequence in the phage genome, known as the protospacer adjacent motif, which is not always available or appropriately positioned. In addition, phages can escape targeting by acquiring mutations in the region recognized by the guide RNA. Finally, the success of the method depends on the precise design of the guide RNA and its ability to bind specifically to the target sequence (Khambhati et al., 2022).

Alternatively, modified phage genomes can be obtained using *in vivo* or *in vitro* genome assembly approaches employing yeast or cell-free systems to assemble long phage genomic DNA, which allows obtaining recombinant phages with the desired modification without going through enrichment and counter-selection steps. Yeast-based assembly has become a widely used strategy for phage genome engineering owing to its ability to efficiently assemble multiple overlapping DNA fragments, its suitability for constructing large genomes that are often difficult to manipulate in bacterial systems and the non-toxicity of the phage genome against yeast (Kilcher and Loessner, 2019). Genome assembly in *Saccharomyces cerevisiae* (a species of budding yeast widely used in biotechnology and genetic engineering) allows for the seamless assembly of multiple DNA fragments, facilitating the creation of full-length phage genomes with desired modifications. These approaches typically involve PCR amplification or *de novo* synthesis of phage genome fragments with overlapping sequences extremities, including one or more fragments containing tailored mutations (see poster, ‘Strategies for phage engineering’). The fragments are then joined in yeast cells via HR. Moreover, genome assembly can also be carried out entirely *in vitro* using solution-based, cell-free systems that replicate cellular functions without the need for a living host organism and allow the precise and rapid joining of DNA fragments (see poster, ‘Strategies for phage engineering’). Once assembled, the phage genome can be extracted and reactivated into infectious phage particles either *in vivo*, by transforming it into host cells or L-forms (bacteria lacking a cell wall, which facilitate the uptake of large DNA molecules), or *in vitro* by using cell-free systems. The *in vitro* approach removes the genome size limitation associated with classical cellular systems, enabling the uptake of large phage genomes and their reactivation into new phage particles, a process often referred to as ‘rebooting’ (Kilcher and Loessner, 2019; Mahler et al., 2023). Rebooting custom-designed synthetic phage genomes offers several advantages, such as precise design and construction of genomes with defined modifications and providing greater flexibility and control over genome content (Assad-Garcia et al., 2022). Among the systems developed to enable this, the use of L-form bacteria for phage genome rebooting represents a pioneering advance in synthetic virology (Kilcher and Loessner, 2019; Assad-Garcia et al., 2022). However, despite their promise, L-form-based rebooting approaches still face several limitations. One of them is the relatively slow kinetics of phage reactivation, likely due to the reduced metabolic activity of L-forms compared to walled bacteria. In addition, although both studies demonstrate that a broad range of phages can be rebooted in L-form systems, the generalizability across phage types and host backgrounds remains to be fully validated. Furthermore, the method depends on specific L-form strains, for which compatibility with diverse phage genomes is not universal.

Taken together, these diverse engineering strategies, from HR and CRISPR-Cas-assisted editing to genome assembly and synthetic rebooting, have greatly expanded the possibilities for precise and efficient bacteriophage modification. These advances lay a strong foundation for unlocking the full therapeutic and biotechnological potential of engineered bacteriophages, which will be explored in the following section.

## Applications of engineered bacteriophages

Phage therapy is one of the most clinically widespread biotechnological uses of phages, owing to its capacity to selectively target multidrug-resistant bacteria, replicate at the infection site and preserve host-associated microbiota, features that are particularly relevant in the context of rising antimicrobial resistance (Strathdee et al., 2023; Ahmad et al., 2024). One key focus in advancing phage therapy is overcoming the limitations of naturally isolated phages (such as their narrow host range, limited biofilm and intracellular efficacy, and inability to counter bacterial resistance mechanisms) through the development of tailored phages (Strathdee et al., 2023). Recent breakthroughs in genetic engineering – such as CRISPR-Cas-assisted genome editing, modular recombineering (i.e. targeted replacement or insertion of functional gene modules using HR) and *de novo* phage genome synthesis (Hussain et al., 2023; Lewis et al., 2024) – have significantly advanced phage engineering techniques, opening up a wide array of applications ranging from novel therapeutics to advanced diagnostic tools and innovative nano-biomaterials.

In this final section we explore the latest applications based on engineered phages.

## Engineered bacteriophages in antibacterial therapy

### Phage host range modulation

In nature, bacteriophages generally target a narrow spectrum of bacterial species. Phage host specificity is primarily determined by the interaction between phage RBPs and bacterial surface receptors (Álvarez-Espejo et al., 2024). Modification of these phage components allows phages to recognize and infect a broader range of bacterial hosts, expanding their host range. The most used techniques for these modifications rely on HR, with or without the use of recombinase-expressing strains, which enables the replacement or fusion of tail fibre genes or receptor-binding domains from different phages to create chimeric structures with expanded specificity (see poster, ‘Technologies based on engineered phages’ panel, ‘Phages as antimicrobial agents’). For example, Trojet and colleagues showed the potential of chimeric tail fibres (tail fibres genetically engineered by combining sequences from different phages) to extend the host range of T4-superfamily phages (long-tailed phages), enabling these phages to infect bacteria across species (Trojet et al., 2011). Although this modularity is well described in the T4-superfamily, applying similar engineering to other phages depends on their RBP architecture and requires detailed structural characterization. Similarly, Cunliffe and colleagues successfully broadened the host range of *Escherichia coli* phage P2 (temperate long-tailed phage) by engineering chimeric tail fibres that incorporate host-specific receptor-binding domains, retargeting toward the OmpC receptor of *Salmonella* (Cunliffe et al., 2022). Comparable results were achieved with *P. aeruginosa* phages PaP1 (long-tailed phage) and JG004 (Le et al., 2013), as well as with a T5-like *Salmonella* phage (Zhang et al., 2022). Using *Listeria* phage PSA as a model, Dunne and colleagues developed a synthetic biology framework for host range engineering. This approach involved identifying and structurally characterizing the RBP Gp15, generating a sequence-randomized RBP library (RBP variants generated by random mutagenesis of gp15), and the production of chimeric phages with predictable and expanded host ranges. Their work highlights the importance of RBP–receptor interactions in enabling the structure-guided engineering of phages with predictable host ranges for therapeutic use (Dunne et al., 2019). Although host range engineering expands the therapeutic potential of phages, this approach remains limited by structural constraints of RBPs and the risk of reduced infectivity or stability in chimeric phages (Kilcher et al., 2018; Strathdee et al., 2023). Nevertheless, engineered phages with broadened specificity offer promising applications against multidrug-resistant pathogens and could support the development of more versatile phage therapy cocktails (Strathdee et al., 2023).

### Conversion of temperate phages into lytic therapeutic phages

Furthermore, the potential of existing therapeutic phages can be expanded by converting lysogenic phages to strictly lytic forms, enhancing their infectivity (Blasco et al., 2019; Bleriot et al., 2024). This strategy is particularly valuable when temperate phages possess interesting host specificity or infectivity traits, but raise concerns associated with lysogeny, such as horizontal gene transfer or the dissemination of virulence factors (Strathdee et al., 2023). By removing key lysogeny-associated genes, these phages can be converted into strictly lytic forms better suited for therapeutic applications (Kilcher et al., 2018). Besides improving safety, this genetic conversion may enhance bactericidal efficacy by maintaining a continuous lytic cycle. However, this approach presents limitations, including the risk of incomplete lysogeny suppression or unintended effects on phage fitness. Despite these challenges, engineered lytic phages hold real promise as therapeutic agents, particularly in the fight against multidrug-resistant bacteria, and offer a way to widen the scope of available phage therapies (Strathdee et al., 2023; Kilcher et al., 2018). A notable example is their compassionate use in the treatment of *Mycobacterium abscessus* infections in cystic fibrosis patients, in whom genetically modified phages lacking lysogeny functions contributed to clinical improvement without the emergence of resistance (Dedrick et al., 2019, 2023).

### Engineered phages with antibacterial payloads

The concept of engineered phages with antibacterial payloads (see poster, ‘Technologies based on engineered phages’ panel, ‘Phages as antimicrobial agents’) aims to overcome limitations associated with traditional phage therapy, such as the emergence of phage-resistant bacteria and narrow host spectrum, by enhancing antimicrobial efficacy through the delivery of heterologous antimicrobial effectors. These recombinant phages can express and release a broad range of bioactive molecules, including biofilm or capsule-depolymerases, cell wall hydrolases, toxins, antimicrobial peptides and CRISPR-Cas systems, thereby improving bacterial clearance even in non-lytic infections or in phage-insensitive populations (Hussain et al., 2023). In a recent study, Du and colleagues notably showed that bacteriophages genetically engineered to carry colicin-like bacteriocins (i.e. small antimicrobial proteins) and cell wall hydrolases can significantly enhance bacterial killing, by up to 2-3 log units of reduction in colony-forming units (CFU), compared to wild-type phages lacking these payloads. These modifications were successfully applied to *E. coli* T7-like lytic phages (short-tailed phages chosen for their compact, well-characterized genome and ease of genetic manipulation). Additionally, the engineered phages helped delay the emergence of bacterial resistance by combining phage-induced lysis with the antimicrobial action of bacteriocins and hydrolases. Furthermore, by equipping phages with distinct effectors, the study enabled the targeting of multiple pathogens within polymicrobial infections (Du et al., 2023). In another study, Park and colleagues engineered a temperate phage to deliver a CRISPR/Cas9 system targeting *Staphylococcus aureus*. This modified phage enabled the removal of major virulence genes from the bacterial chromosome (Park et al., 2017). Lu and Collins engineered *E. coli* T7 phage (short-tailed phage) to express the biofilm-degrading enzyme dispersin B during infection, enabling the simultaneous targeting of bacterial cells and the biofilm matrix (Lu and Collins, 2007). The same authors showed that the phage M13 (filamentous phage) could be genetically modified to deliver a gene encoding a non-cleavable variant of the LexA repressor, which inhibits the bacterial SOS response, a global regulatory network activated upon DNA damage and also involved in the induction of certain virulence factors, such as Shiga toxin (Stx) (Lu and Collins, 2009). This inhibition sensitized *E. coli* to antibiotics such as ciprofloxacin and ampicillin, significantly enhancing antibiotic effectiveness, defined here as a reduction in bacterial survival by up to sevenfold compared to that from antibiotic treatment alone. Wang and colleagues reviewed recent advances in the use of engineered phages for biofilm control and emphasized the potential use of genetically modified phages to improve biofilm eradication by producing cell wall- and biofilm matrix-degrading enzymes and displaying biofilm-targeting peptides (Wang et al., 2023). In addition to delivering antibacterial payloads, engineered bacteriophages can be modified to display nanoparticle-binding peptides on their capsids. This strategy enables the controlled conjugation of antimicrobial nanoparticles, such as silver, enhancing phage antibiofilm activity and delaying biofilm regrowth (Szymczak et al., 2024).

Although these studies highlight the therapeutic potential of engineered phages carrying antibacterial payloads, the extension of these approaches to different phage types remains challenging owing to genetic constraints (such as limits on genome size or gene packaging capacity) and the need to preserve phage replication efficiency after modification (to avoid loss of infectivity or propagation) (Kilcher et al., 2018; Strathdee et al., 2023).

### Targeting intracellular bacteria

Treating intracellular bacterial pathogens poses a significant challenge because they are protected from extracellular immune responses and many traditional antibiotics (Fraunholz and Sinha, 2012; Bongers et al., 2019). Intracellular bacteria can disseminate systemically by exploiting host cells as ‘Trojan horses’. Upon lysis of the host cell, the bacteria are released, allowing them to colonize new tissues and establish new foci of infection. Effectively targeting and eliminating these intracellular pathogens, which include *S. aureus*, *E. coli*, *Listeria monocytogenes*, *Mycobacterium tuberculosis* and *Chlamydia trachomatis*, remains a critical challenge in combating infectious diseases (Bongers et al., 2019). Although phages are traditionally defined as viruses that infect bacteria, some have been shown to enter mammalian cells through non-specific uptake mechanisms such as macropinocytosis or endocytosis (Bodner et al., 2021). Although this internalization suggests potential for targeting intracellular pathogens, studies have shown that phages do not always reach the same intracellular compartments as the bacteria they aim to eliminate, which may limit their therapeutic efficacy (Bichet et al., 2021; Bodner et al., 2021). To overcome these limitations, phages can be modified using molecular engineering approaches designed to enhance their cellular uptake and direct them toward specific intracellular compartments. Bhattarai and colleagues modified M13 phage to carry an integrin-binding peptide (RGD), which significantly reduced intracellular *C. trachomatis* in HeLa 229 cells, a human epithelial cell line chosen for its relevance to the genital tract, the primary site of chlamydial infection in humans (Bhattarai et al., 2012). More recently, Williams and colleagues developed phage-based strategies to target intracellular pathogens, based on the genetic engineering of phage K1F (short-tailed phage) to display human epithelial growth factor, thereby enhancing uptake by epithelial cells and enabling the phage to target intracellular *E. coli* K1 (a clinically relevant encapsulated strain) in different human cell lines. To test this approach, they used HeLa cells and hCMEC/D3 cells, two human cell lines that model epithelial and endothelial barriers relevant to the infection and dissemination of *E. coli* K1 (Williams et al., 2023). Additionally, Zhao and colleagues demonstrated enhanced cellular uptake and improved inhibition of intracellular *Salmonella* infections, using *Salmonella* phages engineered to display cell-penetrating peptides. These peptides facilitate the transport of phages across the plasma membrane by promoting cellular internalization, thereby allowing the phages to reach and kill intracellular bacteria (Zhao et al., 2023). These studies highlight the potential of phages engineered with surface-displayed peptides to facilitate penetration of mammalian cells and intracellular bacterial targeting (see poster, ‘Technologies based on engineered phages’ panel, ‘Phages as antimicrobial agents’, ‘Targeting intracellular bacteria’). One of the main challenges of these approaches is that intracellular pathogens occupy different compartments within host cells, such as the cytosol, phagosomes or specialized vacuoles. This diversity complicates the design of phages capable of reaching and eliminating bacteria in their specific intracellular niches.

Together, these approaches illustrate how engineered bacteriophages can be tailored to address key challenges in antibacterial therapy, whether by expanding host range, enhancing lytic activity, delivering antibacterial payloads or improving intracellular targeting. Although each strategy presents specific limitations, their development opens promising avenues for tackling multidrug-resistant infections. However, the clinical translation of genetically engineered bacteriophages raises specific regulatory and safety challenges beyond those encountered with wild-type phages. As genetically modified organisms, engineered phages are subject to stringent biosafety and regulatory frameworks that govern their production, environmental release and clinical use (Kilcher et al., 2018; Hussain et al., 2023). Concerns include the stability of genetic modifications, potential off-target effects, risks of horizontal gene transfer and unintended ecological impacts. Addressing these challenges through rigorous scientific evaluation and harmonized regulatory approaches will be essential to ensure the safe and responsible clinical application of engineered bacteriophages. In the next section, we explore how engineered phages are also emerging as powerful tools for bacterial detection and diagnostic applications.

## Engineered bacteriophages as diagnostic tools

### Reporter phages

The application of engineered reporter bacteriophages (phages that emit a detectable signal upon infecting bacteria) represents an interesting approach for the rapid, accurate and specific detection of bacterial pathogens in clinical samples, such as body fluids and patient-derived secretions. These reporter phages are genetically modified to produce measurable signals during infection of their target bacteria. Depending on the reporter gene inserted inside phage genome, the signal can be bioluminescence (Fernbach et al., 2025), fluorescence (Møller-Olsen et al., 2018) or colorimetric (Chen et al., 2017) (see poster, ‘Technologies based on engineered phages’ panel, ‘Phages as diagnostic tools’). Wheatley and colleagues demonstrated the potential of engineered reporter phages for the rapid detection of *E. coli* in human urine samples from patients with suspected urinary tract infections (Wheatley et al., 2024 preprint). The phages were genetically modified to carry a luciferase reporter gene, enabling bioluminescent signal production upon infection of viable *E. coli* cells. Their approach was validated *in vitro* and *ex vivo*, highlighting its relevance for point-of-care diagnostics in human healthcare (Wheatley et al., 2024 preprint). Brown and colleagues developed modified staphylococcal phages expressing NanoLuc luciferase for detection of pathogens in clinical samples, such as blood, nasal swabs and wound exudates (Fernbach et al., 2025). Similarly, Meile and colleagues developed NanoLuc-based reporter phages to detect the most prevalent urinary tract infection pathogens – *E. coli*, *Klebsiella* spp. and *Enterococcus* spp. – and demonstrated their application in human urine samples (Meile et al., 2023), highlighting the promise of reporter phages in clinical diagnostics. Using reporter phages in clinical diagnostics offers several practical advantages over traditional detection methods. Unlike standard cultures, which can take up to 48 h, reporter phages produce results within a few hours, sometimes as fast as 30 min (Meile et al., 2023; Wheatley et al., 2024 preprint). They also differ from molecular methods such as PCR in that they only detect live, metabolically active bacteria, which helps avoid false positives due to residual DNA from dead cells (Wheatley et al., 2024 preprint). The limit of detection can be quite low, around 10² to 10³ CFU/ml in optimal conditions, making them sensitive enough for clinical use (Brown et al., 2022; Meile et al., 2023). That said, there are still limitations. In some cases, especially in complex samples such as blood or urine, components in the sample can interfere with signal production or phage activity. In addition, because phages typically have a narrow host range, not all clinical strains are susceptible to a given reporter phage, which may limit diagnostic coverage and affect test sensitivity (Meile et al., 2023; Brown et al., 2022).

### Phage-based biosensors

Genetically engineered M13 phages have emerged as valuable tools in biosensor development (see poster, ‘Technologies based on engineered phages’ panel, ‘Phages as diagnostic tools’). Their ability to display target-specific peptides and their high surface area for functionalization allows these phages to act as recognition elements in a variety of sensor platforms. In colorimetric sensors, engineered M13 phages displaying metal-binding peptides have been used to detect mercury ions (Hg²^+^) with a detection limit as low as 80 nM (Wang et al., 2017). This is close to the US Environmental Protection Agency regulatory limit for mercury in drinking water (10 nM), making the method potentially relevant for environmental monitoring. The sensor was tested in aqueous buffer solutions, although its performance in complex environmental samples remains to be validated (Wang et al., 2017). Similarly, phage–gold nanoparticle (AuNP) hybrid networks enabled visual detection of bacterial pathogens such as *E. coli* and *P. aeruginosa* in diverse samples, including seawater and human serum, using engineered M13 phages displaying bacterial-binding peptides. This visual readout is based on a colour shift resulting from changes in localized surface plasmon resonance (LSPR) that occur when gold nanoparticles aggregate around bacterial targets, altering their interaction with light (Peng and Chen, 2019). For virus detection, sandwich-like assemblies using M13 phage, magnetic nanoparticles and AuNPs enabled colorimetric detection of avian influenza virus (H5N1) at 50 plaque-forming units (PFU)/ml in spiked chicken serum. In this design, phage–AuNP conjugates and antibody-coated magnetic nanoparticles co-bind the viral target, leading to particle aggregation and a visible colour change due to LSPR effects. The assay provides a rapid, specific and equipment-free alternative to traditional virological diagnostics, and its detection limit is comparable to that of standard PCR-based assays (Xu et al., 2021). In electrochemical sensors, phages displaying human serum albumin (HSA)-binding peptides have been integrated into poly 3,4-ethylenedioxythiophene (PEDOT) films for the specific detection of HSA, a key biomarker of liver and kidney function. The sensor demonstrated high sensitivity, with a detection limit of ∼500 nM in synthetic urine, which is clinically relevant for detecting abnormal HSA levels in early-stage kidney disease (Ogata et al., 2017).

M13 phages have also been used in surface-enhanced Raman scattering (SERS)-based sensors, in which silver nanowire substrates were chemically functionalized with phages displaying paraquat-binding peptides to enhance Raman signal detection of highly toxic pesticides such as paraquat (Koh et al., 2018). SERS is a sensitive analytical technique that amplifies Raman scattering signals using metallic nanostructures, enabling ultra-trace detection of target molecules through enhanced electromagnetic fields at the substrate surface. These sensors maintained their detection performance in the presence of varying pH and ionic conditions, supporting their potential for practical applications (Koh et al., 2018). More recently, phage-based biosensors have been explored for cancer detection. A notable example is the bifunctional M13 phage biosensor developed by Juusti and colleagues for classifying metastatic urological cancers from urine. These biosensors use phages selected for their affinity to metastasis-associated biomarkers, with detection relying on changes in liquid crystalline behaviour and optical properties upon biomarker binding. This method showed significant potential for non-invasive cancer screening and highlights the emerging role of phage biosensors in oncology diagnostics (Juusti et al., 2024).

Altogether, these advances show how engineered bacteriophages can serve as highly versatile diagnostic tools; whether as reporter phages for rapid detection of live pathogens or as functionalized biosensors for environmental, medical or even oncological applications, offering flexible, sensitive and practical solutions that complement or improve on conventional methods.

## Engineered bacteriophages as nano-biomaterials for eukaryotic applications

### Tissue engineering and regenerative medicine

Engineered phages are increasingly utilized as scaffolds for assembling intricate nanostructures, particularly in tissue regeneration applications. Filamentous phages are often used for these applications because of their nanofibre-like morphology and a highly ordered arrangement of coat proteins, which allows them to self-assemble into ordered scaffolds and display multiple signalling peptides (Cao et al., 2019). These features make them promising tools for promoting tissue regeneration. However, challenges remain, such as controlling the precise assembly of nanostructures and ensuring phages' stability in physiological environments. Peptides selected through phage display have been utilized to promote the regeneration of diverse tissues, including bone, nerves, cartilage, skin and cardiac tissue (Hefferon et al., 2024 preprint). For bone regeneration, phages have been integrated into biomaterial scaffolds to promote osteogenesis and angiogenesis (see poster, ‘Technologies based on engineered phages’ panel, ‘Tissue engineering and regenerative medicine’). In a rat radial bone defect model, RGD-modified M13 phages integrated into hydroxyapatite and chitosan scaffolds, combined with mesenchymal stem cells, enhanced vascularized bone formation compared to scaffolds containing wild-type phages or no phages. After 8 weeks, increased bone volume density and a higher number of newly formed blood vessels were observed in the RGD–phage group (Wang et al., 2014). Electrospun polycaprolactone scaffolds coated with alginate and RGD-modified M13 phages chemically conjugated via a cross-linking agent showed significantly improved *in vitro* osteogenic outcomes, such as enhanced mineralization by osteoblast-like cells, compared to scaffolds coated with physically mixed M13 phage/alginate or RGD-modified alginate alone (Lee et al., 2016). DPI-VTK, a dual-function peptide identified by phage display, was used to mimic bone matrix and supported osteogenesis and vascularization *in vivo* in a subcutaneous implantation model in mice, at a level comparable to that of clinical collagen-based peptide P15 (Ramaraju and Kohn, 2019). Phages can also be applied in skin regeneration and may be particularly effective for treating infected or chronic wounds, where they support tissue repair while also providing antibacterial protection. M13 phages displaying RGD and IKVAV peptides (the latter being a laminin-derived motif promoting cell adhesion and migration) have been shown to promote fibroblast adhesion and proliferation on engineered scaffolds *in vitro*, suggesting potential for applications in wound healing (Shin et al., 2014). M13 phage matrices, both with and without RGD modification, were arranged into nano-in-micro ridge structures via dip-pulling, a one-step immersion and withdrawal process that aligns phage nanofibre bundles into ordered microstructured patterns. These matrices promoted the co-differentiation of induced pluripotent stem cell-derived neural stem cells into neurons and astrocytes *in vitro*, a desired outcome as both cell types are essential for recreating the complex architecture and supportive environment of neural tissue, which is critical for successful tissue repair (Zhou et al., 2019). In ischemic tissue repair, RGD-modified M13 embedded in polyacrylamide hydrogel with endothelial progenitor cells promoted angiogenesis and antioxidative effects in affected regions (Shrestha et al., 2022). Despite the progress made in this direction, the clinical translation of engineered phages in tissue engineering remains limited, partly owing to their short *in vivo* half-life and rapid clearance by the immune system, which tends to eliminate them through phagocytosis or neutralizing antibodies, and partly because of regulatory constraints that affect both wild-type and genetically modified phages (Pirnay et al., 2018).

### Phage-mediated drug and gene delivery

Current drug administration routes, including oral, gastrointestinal and transdermal methods, often face limitations in therapeutic efficacy owing to physiological barriers that hinder absorption. However, increasing drug dosage to maximize uptake can result in systemic exposure, which in turn can lead to off-target distribution and high toxicity. Advanced drug delivery systems aim to optimize therapeutic efficacy by enabling intracellular and site-specific delivery, addressing several challenges related to drug targeting, toxicity, stability, solubility and bioavailability. Genetically engineered phages, either alone or in combination with nanocarrier systems, have emerged as promising carriers for both drug and gene delivery. Similar to phage display technology, this strategy combines the high binding selectivity of engineered phages with the therapeutic potential of payloads to achieve precise drug delivery to pathogenic or cancerous cells (see poster, ‘Technologies based on engineered phages’ panel, ‘Phages as delivery systems’) (Manivannan et al., 2022). Phage-mediated delivery involves inserting a therapeutic gene into a phage genome or attaching nanoparticles (such as drug-loaded liposomes or metallic nanoparticles) to the surface of engineered phages (Fakhiri and Grimm, 2021; Ju and Sun, 2017). These phages are then targeted to specific cells, delivering the payloads directly to the site of action (Hussain et al., 2023). For example, localized energy deposition strategies, such as photothermal and photodynamic therapies, have shown promise in cancer treatment. Photothermal therapy leverages near-infrared wavelengths and photothermal agents (such as AuNP-modified phages conjugated with gold nanorods) to induce externally controlled hyperthermia for targeted cancer cell ablation. This effect was demonstrated *in vitro* on prostate cancer cells, with treatment success assessed by reduced cell viability after irradiation (Oh et al., 2015). Photodynamic therapy utilizes photosensitizers to generate cytotoxic singlet oxygen upon light activation, selectively destroying cancer cells. Engineered M13 phages conjugated with Chlorin e6 or Rose Bengal, two well-known photosensitizers, have notably shown efficacy in targeting ovarian and colorectal cancer cells, promoting reactive oxygen species production and reduced viability after irradiation (Bortot et al., 2022; Turrini et al., 2024). Phage display has also been explored in a therapeutic context for Alzheimer's disease and for drug delivery into the brain. In Alzheimer's disease, M13 phages engineered to display amyloid-β-derived peptides have been used to detect soluble amyloid-β oligomers in brain tissue, both in transgenic mouse models and human post-mortem samples (Martins et al., 2024). Although phage-based delivery systems show exciting potential, several hurdles remain. Low uptake by mammalian cells, endosomal trapping and inefficient nuclear targeting still limit their effectiveness for gene delivery.

### Phage-based vaccination strategies

Phage-based vaccination represents an innovative approach to vaccine development that leverages the ability of phages to display specific antigens and induce protective immune responses. Phages can be genetically engineered to display a wide range of epitopes derived from viral, bacterial and tumour-associated antigens, making them highly versatile platforms for vaccine development (see poster, ‘Technologies based on engineered phages’ panel, ‘Phage-based vaccines’). They offer several advantages, including their potential for low cost; relative simplicity to prepare and to genetically modify; scalable production; resistance to nuclease, protease and desiccation; adjuvant capacity; high antigen display density; ability to deliver one or more eukaryotic expression cassettes encoding antigens simultaneously; and stimulation of both humoral and cellular immune responses (mainly shown in animal models) (Bao et al., 2019; González-Mora et al., 2020). For example, Hashemi and colleagues developed filamentous phages displaying the herpes virus glycoprotein D, which elicited comparable humoral and cellular immune responses in mice, highlighting their potential as alternative vaccine platform (Hashemi et al., 2010). Although these results were obtained in mice, a standard preclinical model for evaluating vaccine immunogenicity, further studies will be needed to confirm their relevance in humans. Beyond antigen display, genetically modified phages demonstrate superior performance in delivering transgenes (foreign genes introduced into cells to produce therapeutic proteins or antigens) and inducing the expression of these proteins within malignant cells, compared to conventional non-viral methods such as liposomes, electroporation or gene gun-based methods (which use high-velocity particles to deliver DNA into cells) (Clark and March, 2006; Bao et al., 2019). Clark and March demonstrated in mice a more significant immune response after vaccination with a λ phage carrying a cassette (under the control of a eukaryotic cytomegalovirus promoter) encoding the hepatitis B virus surface antigen (HBsAg) compared to vaccination with naked DNA (Clark and March, 2004). Davenport and colleagues showed that phage-like particles derived from phage λ, chemically linked to viral antigens, could trigger strong neutralizing antibody responses and protect animals against infection by severe acute respiratory syndrome coronavirus 2 (SARS-CoV-2) or Middle East respiratory syndrome coronavirus (MERS-CoV) (Davenport et al., 2022). Phages have also been used to deliver DNA vaccines or genetic adjuvants. This promotes intracellular antigen expression and presentation via both major histocompatibility complex (MHC) class I and II pathways, and ultimately triggers robust both innate and adaptive immune responses, which result from their intrinsic ability to interact with pattern recognition receptors (such as TLR9) and to facilitate antigen uptake, processing and cross-presentation by antigen-presenting cells (Hess and Jewell, 2020). Preclinical studies have shown that DNA delivered by phages can target antigen-presenting cells, enabling cytoplasmic and in some cases nuclear, entry of the genetic material, which supports sustained antigen expression. This strategy enhances both cytotoxic T-lymphocyte responses and antibody production, combining the delivery capacity and adjuvant properties of the phage particle. Overall, the applications discussed in this section, ‘Engineered bacteriophages as nano-biomaterials for eukaryotic applications’, highlight the versatility of phages as platforms for tissue regeneration, targeted delivery and vaccination strategies. Their capacity for functionalization and biological compatibility supports a wide range of biomedical uses. However, clinical translation remains limited by challenges such as *in vivo* stability, immune clearance and regulatory hurdles.

## Conclusions

In this At a Glance article, we examine the expanding role of engineered phages in addressing critical challenges in health and biotechnology, particularly in the context of rising antibiotic resistance. By highlighting advanced strategies for phage engineering to enhance their potential as antibacterial agents and exploring the use of phage display to create specific peptides, diagnostic tools, targeted drug delivery systems and nano biomaterials, we provide a comprehensive overview of innovative phage engineering strategies to extend their natural capabilities toward diverse therapeutic applications. The potential of engineered phages as versatile tools in a rapidly evolving healthcare landscape stresses the need for continued research and development to fully realize their therapeutic and biotechnological potential. It is now, more than ever, prominent that phage engineering can be a key solution and the future to solving the problem of antimicrobial resistance, especially when it comes to personalized medicine. Various limitations related to the use of natural phages – including the risk of immune response, difficulty in determining clinical doses, rapid toxin release and the fact that only lytic phages can be used for phage therapy – can be overcome by the use of genetically modified phages. Genetically modified phages, when combined in cocktails or with antibiotics, can target the most difficult-to-treat infections, including those caused by intracellular bacteria. This brings a novel growing field of research, the study of molecular mechanisms of phage therapy at a cellular level, including the investigation of phage–human cell interactions and their effect on clearing bacterial infections. Advances in synthetic biology and phage genetic modifications can aid in the study of these mechanisms, which are essential to explore in depth to advance phage therapy. The integration of AI and bioinformatics in phage design is equally essential and will further boost personalized phage therapy. These can be tools for the prediction of accurate genetic modification, e.g. of phage tail fibres or of bacterial receptors, to expand the phage tropism or create ‘phage-multi tools’ that can target more than one bacterial species. Overall, the collaboration of different disciplines, including phage biologists, molecular biologists, synthetic biologists, microbiologists, cell biologists, evolutionary biologists, immunologists, clinicians, bioinformaticians, chemists and computer scientists can help phage platforms to be developed and accelerate the accuracy and safety of modified phages for therapy. Finally, phage therapy aligns with One Health approaches, which advocate for new strategies to combat global public health challenges that consider the interconnectedness of human, animal and environmental health.

## Poster

Poster
