# Supplementary figures and images for: Engineered bacteriophages for therapeutic and diagnostic applications
Source: Dis Model Mech. 2025 Sep 30;18(9):dmm052393. doi: 10.1242/dmm.052393 (PMC12519544; doi:10.1242/dmm.052393)

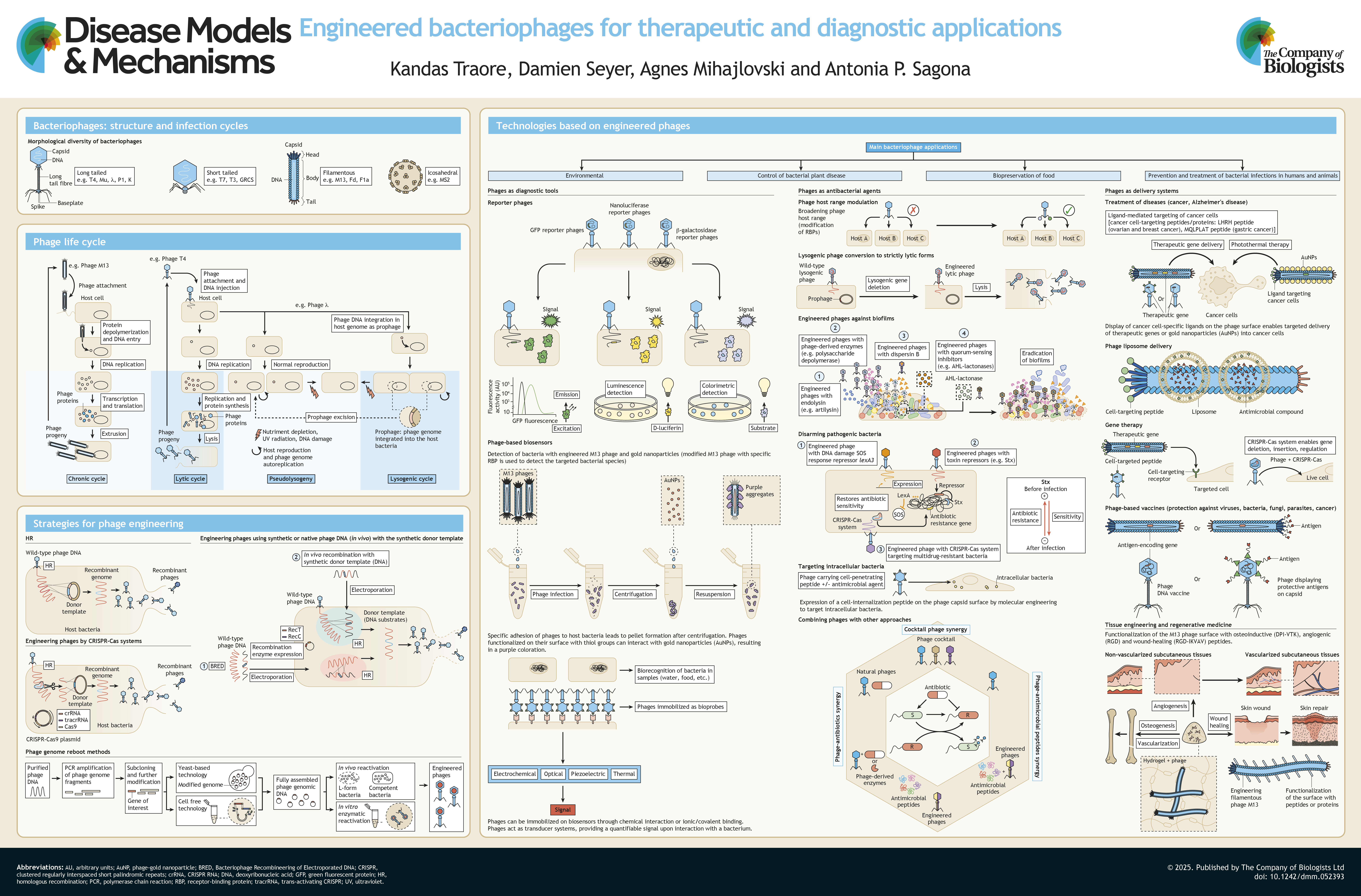

Supplement: Poster [file dmm-18-052393-s1.jpg]
